# Novel Bioactive Kefiran-Based Films Enriched with Grape Pomace Extract

**DOI:** 10.3390/polym17233108

**Published:** 2025-11-23

**Authors:** Rosalba Paola Islas-Enríquez, Julia M. Márquez-Reyes, Juan G. Báez-González, Sergio A. Galindo-Rodríguez, Claudia T. Gallardo-Rivera, Ezequiel Viveros-Valdez, Carlos Abel Amaya-Guerra, Minerva Bautista-Villarreal, Mayra Z. Treviño-Garza

**Affiliations:** 1 Facultad de Ciencias Biológicas, Universidad Autónoma de Nuevo León, Av. Pedro de Alba S/N, Cd. Universitaria, San Nicolás de los Garza 66455, Mexico; rosalba.islase@uanl.edu.mx (R.P.I.-E.); juan.baezgn@uanl.edu.mx (J.G.B.-G.); sergio.galindord@uanl.edu.mx (S.A.G.-R.); claudia.gallardorv@uanl.edu.mx (C.T.G.-R.); jose.viverosvld@uanl.edu.mx (E.V.-V.); carlos.amayagr@uanl.edu.mx (C.A.A.-G.); minerva.bautistavl@uanl.edu.mx (M.B.-V.); 2 Facultad de Agronomía, Universidad Autónoma de Nuevo León, Francisco I. Madero S/N, Ex Hacienda el Cañada, Escobedo 66050, Mexico; julia.marquezrys@uanl.edu.mx

**Keywords:** kefiran, edible films, grape pomace, phenolic compounds, active packaging

## Abstract

The increasing demand for eco-friendly and functional packaging materials has driven research on biodegradable materials incorporating bioactive compounds. In this study, kefiran-based films (K; 3%) were developed and incorporated with grape pomace extract (GPE) at different concentrations (3K-0.5GPE, 3K-1.0GPE, and 3K-1.5GPE). The films were characterized based on their physicochemical, mechanical, antioxidant, and antimicrobial properties. It was found that the incorporation of GPE into the films increased the L*, a*, b*, and Δ*E* values, as well as the thickness, and improved UV radiation protection. FT-IR analysis revealed interactions between kefiran and the phenolic compounds of GPE, without altering the polymer structure. In addition, an increase in tensile strength and elongation at break was observed, evidencing a plasticizing effect of GPE, which also increased the water vapor permeability of 3K-1.5GPE. Solubility was not affected by the incorporation of GPE into the films. Regarding bioactive properties, the addition of GPE increased antioxidant activity and total phenolics. Antimicrobial assays showed activity only for the 3K-0.5GPE film against *Listeria monocytogenes*, with no activity against *Escherichia coli*. Overall, kefiran-based films containing GPE exhibit characteristics that position them as potential alternatives for sustainable, bioactive food packaging materials, thereby promoting the valorization of by-products from the wine industry.

## 1. Introduction

At present, one of the greatest challenges in the food industry is food preservation driven by the growing demand for sustainable alternatives that meet food safety requirements. Proper food preservation is essential to maintain the quality and safety of products throughout the supply chain. This often involves the use of conventional plastic packaging, which, although effective, causes serious environmental problems due to its long-term persistence, resulting in harmful effects on marine and terrestrial ecosystems [1,2]. Some reports estimate that the production of synthetic plastics reaches about 200 million tons per year [3], of which approximately 34 million enter aquatic ecosystems [4]. These materials can persist in the environment for long time periods and pose a severe threat to biodiversity [5,6,7].

To address the environmental issues associated with conventional plastics, multiple studies have focused on the development of new biodegradable packaging materials derived from renewable sources. These materials include biopolymers such as proteins (e.g., gelatin, casein, whey protein, and soy protein), lipids (beeswax, carnauba wax, fatty acids, and acylglycerols), and polysaccharides (chitosan, starch, pectin, carboxymethylcellulose, and alginate), either as individual components or in combination [8,9,10]. Furthermore, polymeric matrices developed from these compounds can be functionalized through the incorporation of antioxidant and antimicrobial agents into the formulations, aiming to produce sustainable packaging capable of providing functional benefits that help extend the shelf-life of food products [11,12].

Kefiran is a microbial exopolysaccharide produced by lactic acid bacteria (LAB), composed of roughly equal amounts of D-glucose and D-galactose and exhibiting a branched structure [13,14,15]. This polymer is obtained during the fermentation of milk in the production of kefir, a fermented dairy beverage whose starter culture consists of irregularly shaped grains formed by a microbial consortium of LAB and yeasts. These grains are held together by kefiran, which protects them from external factors such as desiccation, nutrient deficiency, and osmotic stress, among others [16]. Kefiran is recognized as safe (GRAS), biodegradable, and biocompatible, with film-forming capacity, excellent mechanical properties, solubility, and gas barrier characteristics [17,18], making it a promising material for food packaging applications.

Some studies have focused on exploring the potential of kefiran in the development of biodegradable packaging materials, either as an individual component [19,20,21,22] or combined with other polymers such as starch [23], chitosan [24], carboxymethylcellulose [25,26], chitosan–nanocellulose [27], whey protein [28,29], water-based polyurethane [30], and *Malva neglecta* mucilage [31], among others. Likewise, the functionalization of kefiran-based films has been reported by incorporating active components, such as the essential oils of *Satureja khuzestanica* [25], *Zataria multiflora*, and *Rosmarinus officinalis* [30], to impart antimicrobial or antioxidant properties that extend the shelf life of various foods. Despite these efforts, the potential of kefiran as a polymeric matrix for active packaging remains poorly explored. Furthermore, since the performance of these materials depends on the type and origin of the active compounds incorporated into the polymer, it is important to investigate different bioactive sources for their functionalization.

Grape pomace is an agro-industrial by-product resulting from wine production, obtained after the pressing of grapes. This by-product, composed of skins, seeds, and stems, represents between 20 and 30% of the total processed grape volume [32]. Its disposal generates environmental problems, such as soil and water contamination, unpleasant odors, and attraction of undesirable fauna, resulting in an imbalance of surrounding ecosystems [33,34]. Grape pomace contains a considerable amount of phenolic compounds, such as anthocyanins, hydroxybenzoic and hydroxycinnamic acids, flavanols, flavonols, and stilbenes, which exhibit notable antioxidant and antimicrobial activity [35,36], making it a promising ingredient for the development of packaging materials with bioactive properties. However, to the best of our knowledge, its incorporation into kefiran-based matrices has not yet been reported.

Several studies have focused on the development of active polymeric films incorporated with grape pomace extract, among which those based on starch/GPE [37], starch/cellulose nanocrystals/GPE [38], chitosan/GPE [39], chitosan/cellulose nanocrystals/GPE [40], sodium alginate/GPE [41,42], pectin/GPE [43], polylactic acid/GPE [44], and polypropylene/GPE [45] stand out. These studies have mainly focused on evaluating the antioxidant and/or antimicrobial properties conferred by the extract, demonstrating its potential for the development of active packaging. However, to the best of our knowledge, the incorporation of GPE into kefiran-based matrices has not yet been reported, representing an opportunity to explore new strategies for the functionalization of this biopolymer for the development of active and sustainable packaging. In this context, the main objective of this research was to develop and characterize kefiran-based films enriched with grape pomace extract, evaluating their physicochemical, mechanical, and bioactive properties for potential use in food preservation and valorizing wine industry by-products.

## 2. Materials and Methods

### 2.1. Materials

#### 2.1.1. Chemicals and Reagents

ABTS radicals (2,2′-azino-bis(3-ethylbenzothiazoline-6-sulfonic acid), purity ≥ 98%) and DPPH radicals (1,1-diphenyl-2-picrylhydrazyl, purity ≥ 98%), as well as Folin–Ciocalteu reagent, potassium persulfate (purity 99.9%), gallic acid (purity 98%), Trolox (6-hydroxy-2,5,7,8-tetramethylchroman-2-carboxylic acid, purity 98.5%), and Tween^®^ 80 were purchased from Sigma-Aldrich^®^ (Toluca de Lerdo, Mexico). Glycerol, ethanol, sodium carbonate, and calcium chloride were obtained from CTR Scientific (Monterrey, Mexico). Brain Heart Infusion (BHI) broth and agar were supplied by BD^®^ Bioxon (Cuautitlán Izcalli, Mexico), and amoxicillin/clavulanic acid (20/10 µg) antibiotic sensitivity discs were obtained from BD^®^ BBL Sensi-Disc (Becton Dickinson, Sparks, MD, USA).

#### 2.1.2. Plant Material

Grape pomace (*Vitis vinifera* L.) from the red Syrah variety was supplied by the winery *Casa Madero*, located in Parras de la Fuente, Coahuila, Mexico, in August 2022. The pomace was transported under refrigerated conditions to the laboratory and stored at –20 °C, protected from light, until further use.

### 2.2. Obtaining of Grape Pomace Extract (GPE)

#### 2.2.1. Preparation of Grape Pomace

The entire grape pomace was used without separating skins, stems, or seeds. The material was dried in a dehydrator (Hamilton Beach 32100A, Glen Allen, VA, USA) at 55 ± 3 °C for 8 h, reaching a moisture content of 6.3 ± 0.13%. Subsequently, it was ground using a coffee grinder (F2034251, Krups, Emmaus, PA, USA), and the resulting powder was sieved to achieve a particle size ≤ 425 µm (sieve No. 40). The pomace powder was stored in polyethylene bags under frozen conditions (−20 ± 2 °C) and protected from light until further use.

#### 2.2.2. Preparation of GPE

The GPE was prepared following the methodology described by Gaber-Ahmed et al. [46] with some modifications. Pomace powder (50 g) was suspended in ethanol at a 1:10 (*w*/*v*) ratio. The mixture was continuously stirred using a magnetic stirrer (Super Nuova, Thermo Fisher Scientific, Waltham, MA, USA) at 600 rpm for 90 min, at room temperature (25 ± 2 °C) and protected from light. Subsequently, the mixture was centrifuged (centrifuge Hermle Z326, Labnet International, Edison, NJ, USA) for 15 min at 9390× *g*. The recovered supernatant was first filtered through Whatman No. 2 filter paper (8 µm) and then through a 0.45 µm nylon membrane filter. The solvent was removed at 45 ± 2 °C under reduced pressure using a rotary evaporator (IKA RV10, Lauda, IKA Works, Inc., Wilmington, NC, USA) coupled to a controlled cooling system (Alpha 8, Lauda, Marlton, NJ, USA). Residual solvent was evaporated in an oven (FE-131, Felisa, Zapopan, Mexico) at 45 ± 2 °C. The resulting dry extract was weighed and stored at −20 °C, protected from light, until further use. The extraction yield (%) was calculated according to the following formula:

$$GPE\;yield\left(\%\right)=\frac{Dry\;extract\;(g)}{Dry\;pomace\;powder\;(g)}\times 100$$

### 2.3. Evaluation of the Antioxidant and Antimicrobial Capacity of GPE

#### 2.3.1. Antioxidant Activity and Total Phenolic Content of GPE

##### Antioxidant Activity by the ABTS Method

The antioxidant capacity of GPE was determined using the ABTS method [47]. To generate the radical, aqueous solutions of ABTS (7 mM) and potassium persulfate (2.45 mM) were first prepared and then mixed in a 1:1 (*v*/*v*) ratio. The mixture was allowed to react for 16 h at 25 ± 2 °C under dark conditions. The resulting ABTS radical solution was diluted with absolute ethanol until obtaining an absorbance of 0.700 ± 0.005 at 734 nm, which was measured using a UV-Vis spectrophotometer (Genesys 10S, Thermo Scientific, Waltham, MA, USA). For the analysis, 300 µL of the sample (appropriately diluted extracts) were mixed with 2700 µL of the adjusted ABTS solution and allowed to react for 7 min at 25 ± 2 °C under dark conditions. The absorbance was then measured at 734 nm, and antioxidant activity was quantified using a calibration curve with Trolox as standard (y = −0.0025x + 0.5804, R^2^ = 0.99). Antioxidant activity was expressed as micromoles of Trolox equivalents per gram of extract (µmol TE/g extract).

##### Antioxidant Capacity by the DPPH Method

Additionally, the antioxidant capacity was evaluated using the DPPH free radical method with some modifications [48]. For the analysis, 750 µL of the diluted extract were mixed with 2250 µL of an ethanolic DPPH solution (0.03 mg/mL, absorbance 1.000 ± 0.005 at 517 nm). The mixture was homogenized using a vortex mixer (Mixer S0200, Labnet International, Edison, NJ, USA) and incubated for 90 min at room temperature (25 ± 2 °C) in the dark to allow the reaction to occur. The final absorbance of the samples was analyzed using a UV-Vis spectrophotometer (Genesys 10S, Thermo Scientific, Waltham, MA, USA) at 517 nm. A calibration curve was prepared using Trolox as the standard (y = −0.0053x + 0.7172, R^2^ = 0.99). Antioxidant activity was expressed as micromoles of Trolox equivalents per gram of extract (µmol TE/g extract).

##### Total Phenolic Content

The total phenolic content was determined using the Folin–Ciocalteu assay with some modifications [49]. A volume of 100 µL of the diluted extract was mixed with distilled water (1500 µL), followed by the incorporation of 100 µL of Folin–Ciocalteu reagent. The mixture was homogenized using a vortex mixer (Mixer S0200, Labnet International, Edison, NJ, USA). Subsequently, 300 µL of sodium carbonate solution (20% (*w*/*v*)) were added, and the mixture was stirred again. The samples were allowed to stand for 90 min at 25 ± 2 °C in the dark. Finally, the absorbance was determined at 760 nm using a UV-Vis spectrophotometer (Genesys 10S, Thermo Scientific, Waltham, MA, USA). Quantification was carried out using a calibration curve (y = 0.0675x − 0.2307, R^2^ = 0.99) with gallic acid as the standard. Results were reported as milligrams of gallic acid equivalents per gram of extract (mg GAE/g).

#### 2.3.2. Determination of Antimicrobial Activity

##### Preparation of Pathogenic Microorganism Inocula

The pathogenic microorganisms *Listeria monocytogenes* ATCC 7644 and *Escherichia coli* (provided by the Department of Immunology and Virology, FCB, UANL) were cultured in 10 mL of Brain Heart Infusion (BHI) broth at 37 ± 2 °C for 16 h. After incubation, the optical density was measured at 625 nm using a UV-Vis spectrophotometer (Genesys 10S, Thermo Scientific, Waltham, MA, USA). The cultures were adjusted to a 0.5 McFarland standard (≈1 × 10^8^ cells/mL) for each microorganism [48].

##### Minimum Inhibitory and Bactericidal Concentrations

The minimum inhibitory concentration (MIC) was determined using the microdilution technique as described by Treviño-Garza et al. [48]. In sterile 96-well plates, 90 µL of BHI broth were added, followed by 100 µL of grape pomace extract at different concentrations (50, 25, 12.5, and 6.25 mg/mL). Subsequently, 10 µL of each pathogen inoculum was incorporated into the wells. The microplates were incubated at 37 ± 2 °C for 24 h. The MIC was determined by the absence of turbidity, indicating inhibition of microbial growth, measured at 625 nm. Based on the MIC results, the minimum bactericidal concentration (MBC) was determined. For this, a 10 µL aliquot from wells showing no visible growth was plated by dropwise inoculation onto BHI agar plates. The plates were incubated at 37 ± 2 °C for 24 h, and the MBC was considered as the lowest concentration showing complete absence of bacterial growth.

### 2.4. Kefiran Production

#### 2.4.1. Kefir Grain Fermentation

The kefiran polymer was obtained through the fermentation of homemade milk kefir grains from Nuevo León, Mexico, which were used as inoculum. Commercial UHT whole milk purchased from local supermarkets served as the fermentation medium. Aseptically, 30 g of kefir grains were placed in sterile 900 mL glass jars with 500 mL of milk. The jars were covered with gauze to allow air exchange and maintained at 28 ± 2 °C for 24 h, without agitation. Daily, the fermented milk was removed using a strainer and replaced with fresh milk to promote microbial biomass growth until the required amount for polymer recovery was achieved [50].

#### 2.4.2. Kefiran Extraction

Kefiran was extracted following the method reported by Sabaghi et al. [24] with some modifications. At the end of the fermentation, the kefir grains were recovered using a strainer, washed with distilled water, and placed in distilled water (1:10 *w*/*v*), maintaining constant boiling and stirring for 1 h. The mixture was centrifuged (Hermle Z326, Labnet International, Edison, NJ, USA) for 20 min at 9390× *g* to recover the supernatant, which was then precipitated with ethanol (1:2 *v*/*v*) at −20 °C for 12 h. In a second centrifugation at 9390× *g* for 20 min, the precipitate (exopolysaccharide) was recovered and dried in an oven at 60 ± 2 °C for 48 h (FE-131, Felisa, Zapopan, Mexico). The kefiran weight was recorded using an analytical balance (XT 220A, Precisa, Dietikon, Switzerland), and the extraction yield (%) was quantified using the following formula:

$$Kefiran\;yield\left(\%\right)=\frac{Dry\;kefiran\;(g)}{Fresh\;kefir\;grains\;(g)}\times 100$$

#### 2.4.3. Kefiran Characterization by Fourier Transform Infrared (FT-IR) Spectroscopy

The dried kefiran was ground in a coffee grinder (F2034251, Krups, Emmaus, PA, USA) to obtain a fine powder, which was characterized using FT-IR spectroscopy on an infrared spectrometer (Frontier, PerkinElmer Inc., Waltham, MA, USA) in the range of 4000–500 cm^−1^ by identifying the bands corresponding to its functional groups [50].

### 2.5. Development of Kefiran Films Incorporated with GPE

#### 2.5.1. Preparation of Film-Forming Solutions

Four film-forming solutions were prepared using 3.0% (*w*/*v*) kefiran. The polysaccharide was dissolved in distilled water at 60 ± 2 °C under constant magnetic stirring (600 rpm). Tween^®^ 80 (0.05% *v*/*v*) and glycerol (0.75% *v*/*v*) were subsequently added into the formulation. Finally, GPE was incorporated at concentrations of 0.5, 1.0, and 1.5% (*w*/*v*). The control formulation consisted of the film without extract (3K). The formulations are summarized in Table 1.

#### 2.5.2. Film Preparation

The films were prepared using the casting method [48]. Film-forming solutions (10 mL) were placed into Petri dishes (60 × 15 mm) and dried in an oven at 42 ± 2 °C for 24 h. The dried films were then carefully removed from the dishes and stored at 50 ± 5% relative humidity, protected from light, until further characterization.

### 2.6. Characterization of Kefiran/GPE Based Films

#### 2.6.1. Evaluation of Physicochemical and Mechanical Properties

##### Color Analysis

Color analysis was performed over the entire surface of the films (60 mm diameter) using a colorimeter (Colorflex^®^ EZ, Hunterlab, Reston, VA, USA), according to the CIELab color system [38]. The coordinates L* (lightness, 0 = black, 100 = white), a* (− = green, + = red), and b* (− = blue, + = yellow) were measured. The total color difference (Δ*E*) between the films containing grape pomace extract and the control film (3K) was calculated using the following equation:

$$\Delta E=\;\sqrt{{\left(L^{*}-L\right)}^{2}+{\left(a^{*}-a\right)}^{2}+{\left(b^{*}-b\right)}^{2}}$$

where L*, a*, and b* represent the color parameters of the developed films, while *L*, *a*, and *b* are the reference values taken from the 3K control film (*L* = 90.09, *a* = −0.66, *b* = −4.22).

##### UV-Vis Barrier Capacity

To evaluate the protective effect of the films against UV-Vis radiation, samples were cut into pieces measuring 30 × 10 mm and analyzed using a UV-Vis spectrophotometer (Genesys 10S, Thermo Scientific, Waltham, MA, USA) in the range of 190–800 nm [51]. The results were expressed as percentage transmittance (%T).

##### FT-IR Spectroscopy Analysis

The films (20 × 10 mm) were analyzed using an FT-IR infrared spectrometer (Perki-nElmer Frontier, PerkinElmer Inc., Waltham, MA, USA) in the range of 4000–500 cm^−1^ to identify the functional groups present [39].

##### Thickness and Mechanical Properties

For thickness determination, the films (60 mm diameter) were measured at 10 points per sample randomly selected across the entire circular surface to ensure coverage of both central and peripheral areas, using a digital micrometer (Coolant Proof IP 65, Mitutoyo USA Inc., Aurora, IL, USA). For mechanical testing, the films were cut into strips measuring 50 × 20 mm. Measurements were performed using a texture analyzer (CT3, Brookfield Engineering Labs Inc., Middleboro, MA, USA) equipped with a tensile grip accessory (TA-RCA). The films were secured between the grips with an initial separation of 10 mm and tested using a 25 kg load cell, an activation load of 1 N, and a speed of 0.5 mm/s. Tensile strength (MPa) and elongation at break (%) were determined using the following formulas [52]:

$$Tensile\;strength\;(MPa)=\frac{Fmax}{A}$$

$$Elongation\;at\;break\;\left(\%\right)=\frac{L_{1}-L_{0}}{L_{0}}\times 100$$

where *Fmax* corresponds to the maximum force before fracture (N), *A* is the cross-sectional area of the film (mm^2^), *L*_1_ is the film length after stretching (mm), and *L*_0_ is the initial length (mm).

##### Water Solubility Evaluation

The solubility of the films was determined following the methodology reported by Sabaghi et al. [24], with some modifications. The films (20 × 20 mm) were dried in an oven at 70 ± 2 °C for 24 h and weighed to obtain their initial dry matter content (w_1_). The dried samples were then placed in distilled water (20 mL) and stirred at 200 rpm for 24 h at 25 ± 2 °C. The resulting samples were recovered by filtration (Whatman No. 1 filter; 11 µm) and dried at 70 ± 2 °C for 24 h to determine the dry matter remaining undissolved in water (w_2_). The percentage (%) solubility of the films was calculated using the following equation:

$$Water\;solubility\left(\%\right)=\frac{w_{1}-w_{2}}{w_{1}}\times 100$$

##### Determination of Water Vapor Permeability (WVP)

The water vapor permeability (WVP) of the kefiran-based films was determined following the methodology reported by Knapp et al. [53]. The films were placed in plastic permeation containers (28 mm in diameter) containing anhydrous CaCl_2_ (1.5 g), which were then sealed. After sealing the containers, they were placed in a desiccator containing a saturated NaCl solution (55 ± 2% relative humidity) and maintained at 25 ± 2 °C for 48 h. The WVP values were determined using the following equation:

$$WVP=\frac{\Delta w}{\Delta t}\times \frac{x}{A\;\Delta P}$$

where Δ*w* represents the weight gain of CaCl_2_ (g), *t* is the elapsed time (days), *x* is the film thickness (mm), *A* is the exposed area (m^2^), and Δ*P* is the partial vapor pressure difference (kPa). The results were expressed in g·mm/m^2^·d·kPa.

#### 2.6.2. Biological Activity of the Kefiran/GPE Films

##### Antioxidant Activity

To evaluate the free radical scavenging activity of the films, 1 g of each film was dispersed in 10 mL of ethanol. The mixture was allowed to rest for 90 min and then centrifuged (centrifuge Labnet Spectrafuge 6C, Labnet International, Edison, NJ, USA) at 4000× *g* for 10 min. The supernatant was analyzed using the ABTS, DPPH, and total phenolic methods, as described in Section 2.3.1. Antioxidant capacity was expressed as µmol TE/g of film, and phenolic content as mg GAE/g of film.

##### Antimicrobial Activity

The antimicrobial activity of kefiran-based films was evaluated using the agar diffusion method [48]. Bacterial strains, *L. monocytogenes* ATCC 7644 and *E. coli*, were cultured as described in Section Preparation of Pathogenic Microorganism Inocula until reaching an optical density of 0.5 on the McFarland scale (≈ 1 × 10^8^ cells/mL). Subsequently, 0.1 mL of each microorganism was inoculated and evenly spread over the surface of ICC agar using sterile swabs. Films, previously cut into 6 mm diameter discs, were placed onto the plates. For the negative control, 6 mm paper discs with 10 µL of isotonic saline solution (0.85%) were used, while for the positive control, 6 mm discs of amoxicillin with clavulanic acid (20/10 µg) were employed. The plates were incubated for 24 h at 37 °C, and inhibition zones were finally expressed in millimeters.

### 2.7. Statistical Analysis

A completely randomized design was employed. Extraction yields of GPE and kefiran were obtained from three independent extractions (*n* = 3). The antioxidant and antimicrobial properties of GPE were carried out in triplicate (*n* = 3). For the films, physicochemical analyses were performed in triplicate (*n* = 3), while mechanical tests were carried out with six replicates (*n* = 6). Antioxidant and antimicrobial properties of the films were also determined in triplicate (*n* = 3). The obtained data were subjected to a normality test (Shapiro–Wilk), followed by analysis of variance (ANOVA) and Tukey’s multiple comparison test (*p* ≤ 0.05), using SPSS Statistics 25 software (IBM Corp., Armonk, NY, USA).

## 3. Results and Discussion

### 3.1. Extraction of GPE

The extraction yield of GPE was 10.42 ± 1.11% using ethanol as the solvent. This result is consistent with that reported by Xu et al. [54], who obtained a yield of 12.4 ± 0.55% for pomace extracts from the Cabernet Franc variety, using an acetone–water (80% *v*/*v*) mixture as the solvent. According to previous studies, the extraction yield is influenced by various factors, such as the nature of the solvent, grape variety, extraction conditions, pomace composition, and pretreatment, among others [49,55], which explains the differences observed among different studies.

### 3.2. Antioxidant and Antimicrobial Activity of GPE

The GPE showed high antioxidant activity, with values of 1081 ± 26.73 µmol TE/g (ABTS) and 819 ± 10.57 µmol TE/g (DPPH), as well as a total phenolic content of 21.65 mg GAE/g (Table 2). These results agree with those reported by Ky and Teissedre [56] for polyphenolic extracts from grape pomace of the Syrah variety obtained with ethanol or hydroalcoholic solutions. Compared with other studies, the antioxidant capacity of our extracts was higher than that reported by Ky et al. [55] for Syrah pomace (83.3–262.9 µmol TE/g ABTS and 49.7–208.4 µmol TE/g DPPH) and by Moutinho et al. [57] for a mixture of pomaces from Touriga Nacional and Sousão varieties extracted with aqueous ethanol (294 and 456 µmol TE/g by ABTS and DPPH, respectively; 44.06 mg GAE/g). Likewise, our results are comparable to those of Xu et al. [54], who obtained 1013 µmol TE/g (ABTS) in pomace from the Cabernet Franc variety using an 80% acetone–water mixture, and to those of Batista et al. [58], who found 644 µmol TE/g (DPPH) in extracts from a mixture of Syrah and Seibel pomaces (75% and 25%, respectively). In contrast, Chengolova et al. [59] reported higher values in seeds of the Cabernet Sauvignon variety extracted with 70% ethanol (2246.23 µmol TE/g by ABTS and 432.25 µmol TE/g by DPPH; 88.22 mg GAE/g), a tissue known to present greater antioxidant capacity than grape skins [60]. In the same study, the skin extracts showed significantly lower activities (99.05 and 81.23 µmol TE/g for ABTS and DPPH, respectively), with a phenolic content of 42.32 mg GAE/g.

In general, the antioxidant capacity observed is related to the phenolic content and profile of the extracts (phenolic acids, flavonols, flavanols, anthocyanins, and stilbenes), which can effectively neutralize free radicals [57]. Cheng et al. [61] note that factors such as grape variety, the type of plant tissue (e.g., stems, seeds, and/or skins), and the extraction solvent used directly influence antioxidant properties. Overall, the use of ethanol as a GRAS solvent provides an adequate balance between efficacy and safety for obtaining phenolic extracts with potential applications in food products.

Regarding the antimicrobial activity, the GPE showed an effect against *L. monocytogenes* and *E. coli*, with *L. monocytogenes* being the most sensitive (MIC: 6.25 mg/mL) compared to *E. coli* (MIC: 50 mg/mL) (Table 2). These results are comparable to those reported by Peixoto et al. [35], who reported minimum inhibitory concentrations (MICs) for *E. coli* ESBL of >20, 20, and 20 mg/mL, and for *L. monocytogenes* of >20, 10, and 20 mg/mL, corresponding to hydromethanolic extracts of skins, seeds, and stems, respectively. Likewise, our results are consistent with those reported by Silva et al. [62], who found MIC values of 25 mg/mL for *E. coli* and 0.78 mg/mL for *S. aureus* in seed extracts of the Pinot Noir variety. Similarly, Grosu et al. [63] obtained MIC and MBC values of 2.23 mg/mL against *L. monocytogenes* in grape pomace extracts of the Fetească Neagră variety, using 50% ethanol as the solvent. In turn, Xu et al. [54] reported MIC values of 4.69 and 18.8 mg/mL for *L. monocytogenes* ATCC 7644 in pomace extracts from the Cabernet Franc and Chambourcin varieties, respectively, without observing antimicrobial activity against *E. coli* O157:H7 ATCC 3510.

According to the literature, the antimicrobial activity of the phenolic compounds present in grape pomace is attributed to their ability to disrupt cell membrane integrity, altering its permeability and causing the loss of intracellular components. They can also interfere with essential enzymes and generate reactive oxygen species that damage structures such as lipids, proteins, and nucleic acids. This effect is more evident in Gram-positive bacteria, such as *L. monocytogenes*, whose cell structure is more vulnerable [64].

### 3.3. Kefiran Production by Fermentation

**Figure 1:**
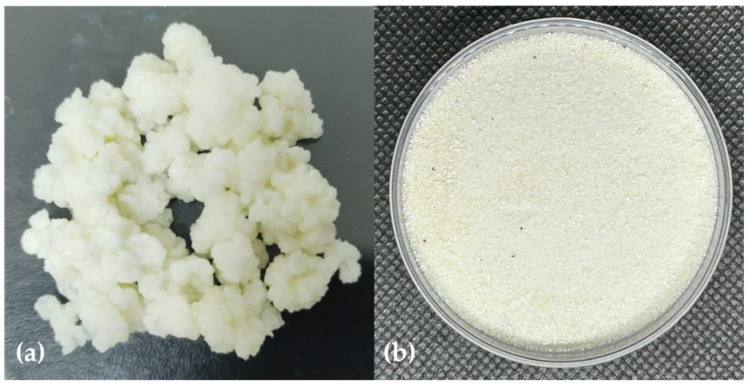
(**a**) Milk kefir grains; (**b**) kefiran, appearing as a pale cream-colored powder, obtained by fermentation.

Figure 1 shows the milk kefir grains (Figure 1a) and the kefiran, which appears as a pale cream-colored powder (Figure 1b). The extraction yield of the polysaccharide on a dry weight basis was 4.94 ± 1.01%, similar to the 4.26% reported by Radhouani et al. [15] and higher than the 2.01% described by Hasheminya et al. [25].

#### Kefiran Characterization by FT-IR

**Figure 2:**
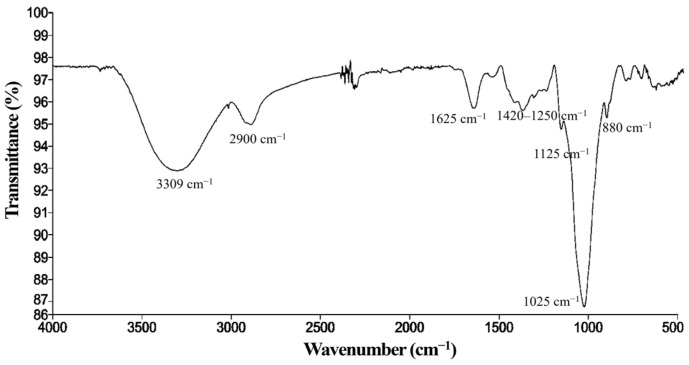
FT-IR spectrum of kefiran.

The FT-IR analysis of kefiran showed characteristic bands providing information about its chemical structure (Figure 2).

The most intense band at 3309 cm^−1^ corresponds to O–H stretching, indicating mainly the presence of hydroxyl groups in kefiran and, to a lesser extent, water adsorbed by the polymer [15,20,50]. At 2900 cm^−1^, the C–H stretching of methyl (−CH_3_) and methylene (−CH_2_^−^) groups is observed [18,20], typical groups in polysaccharides [65]. The signal at 1625 cm^−1^ is associated with the H–O–H bending of water bound in the exopolysaccharide [20,66]. The bands in the 1420–1250 cm^−1^ region can be attributed to C–H and O–H groups [50]. Intense absorptions were recorded at 1125 cm^−1^ and 1025 cm^−1^, characteristic of C–O–C bonds, which are fundamental in carbohydrate structures [18,65]. Finally, the signal at 880 cm^−1^ corresponds to β-glycosidic bond vibrations, indicating the presence of a monosaccharide backbone, typically glucose and galactose, within the structure of kefiran, confirming that the obtained compound is a polysaccharide [15]. Overall, the FT-IR spectrum evidences a material composed mainly of carbohydrates with glycosidic bonds and hydroxyl groups, suitable as a base material for film development [67].

### 3.4. Development of Kefiran-Based Films with Grape Pomace Extract

**Figure 3:**
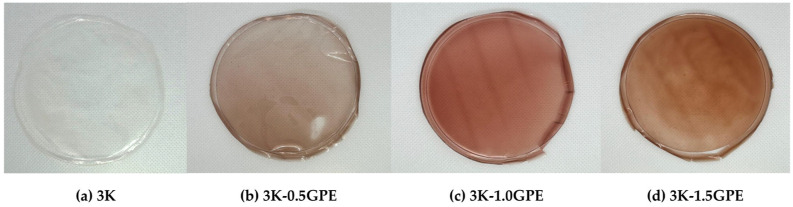
Kefiran-based films containing grape pomace extract at different concentrations (0, 0.5, 1.0, and 1.5%).

Figure 3 shows the films obtained from kefiran (3K) and with the addition of grape pomace extract at different concentrations (0.5, 1.0, and 1.5% *w*/*v*). The films obtained were homogeneous, flexible, and elastic, with a smooth surface and slight gloss. The control (3K) was colorless and transparent, whereas in the formulations containing the extract, the intensity of reddish tones and the opacity increased with the GPE concentration. Similar results have been reported in chitosan nanocomposite films incorporating cellulose nanocrystals and GPE [40] and arrowroot starch films incorporated with GPE [68].

### 3.5. Characterization of the Kefiran/GPE-Based Films

#### 3.5.1. Physicochemical and Mechanical Properties

##### Color

In general, the color of the films depended on the extract concentration. The incorporation of GPE significantly (*p* ≤ 0.05) affected the color parameters L*, a*, b*, and Δ*E* of the kefiran films (Table 3). Luminosity (L*) decreased from 90.09 ± 0.64 in the control (3K) to 55.07 ± 1.67 in the film with the highest extract concentration (3K-1.5GPE). The a* coordinate increased from −0.66 ± 0.03 (3K) to 1.70 ± 0.09 (3K-0.5GPE), and as the extract concentration increased, higher values were observed, similar for 3K-1.0GPE (4.47 ± 0.08) and 3K-1.5GPE (4.20 ± 0.18), showing a trend toward redder tones with the addition of GPE; this effect is associated with the color contribution of phenolic compounds, mainly anthocyanins, as well as flavonols and tannins [41,43].

Furthermore, the b* values increased significantly, from −4.22 ± 0.21 (3K) to 2.46 ± 0.15 (3K-1.5GPE), indicating a trend toward yellowish tones. These findings are consistent with those reported by Xu et al. [40] and Etxabide et al. [69], who observed a decrease in L* values and an increase in a* and b* values when raising the concentration of grape pomace extracts in chitosan/cellulose nanocrystal films and gelatin films, respectively. Finally, the Δ*E* values of the films incorporated with GPE relative to the control (3K) increased as the extract concentration increased (15.11–35.98; Table 3), indicating a perceptible change in film color, in accordance with Mileti et al. [70] in starch films with anthocyanins from pomace and red cabbage. Since color affects consumer perception of quality in packaged products [71], these results highlight the effect of GPE on the optical properties of kefiran films.

##### UV–Vis Protection

**Figure 4:**
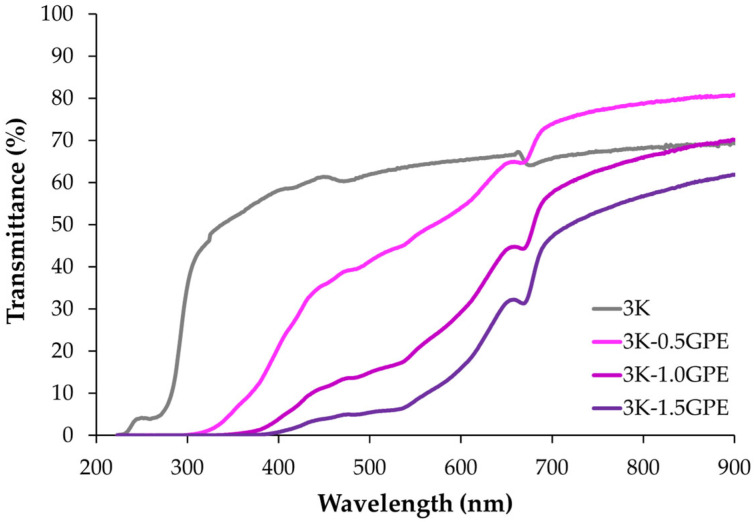
Protective effect of kefiran-based films containing GPE (0, 0.5, 1.0, and 1.5%) against UV–Vis radiation.

Kefiran films incorporated with GPE showed a decrease in UV–Vis transmittance values (Figure 4), particularly in the ultraviolet region (200–400 nm). As the extract concentration increased, the UV-blocking capacity improved relative to the control, with this effect being more pronounced in the 3K-1.0GPE and 3K-1.5GPE formulations. Likewise, in the visible region (400–700 nm), increasing the GPE concentration reduced transmittance; this behavior is associated with increased opacity and lower visible light transmission as the concentration of phenolic compounds in the films rises [69,72]. These compounds (e.g., anthocyanins, flavonols, and tannins) have conjugated structures capable of absorbing radiation in the UV–Vis region, acting as natural filters and reducing the amount of light passing through the films [73,74].

The results observed in kefiran-based films with GPE are consistent with those reported by Gubitosa et al. [42], who found the same trend in sodium alginate films with aqueous grape pomace extract, observing a progressive decrease in UV transmittance as the extract concentration increased (10, 20, and 40%). Similarly, Ji et al. [75] reported UV-blocking values close to 100% in cellulose films containing grape seed extract. Likewise, da Silva et al. [45] observed that polypropylene films incorporated with grape pomace extracts blocked transmittance in the UV region (200–300 nm). This attenuation of radiation, particularly in the UV range, is desirable in food packaging materials, as it protects photosensitive components without excessively compromising transparency in the visible region [76].

##### FT-IR Spectroscopy

**Figure 5:**
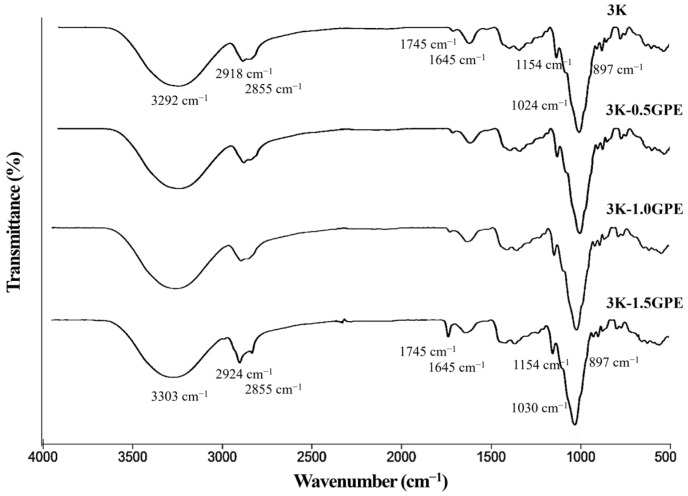
FT–IR spectrum (4000–500 cm^−1^) of kefiran-based films incorporating grape pomace extract (0, 0.5, 1.0, and 1.5%).

In general, the FT-IR spectra of the films were similar across treatments (Figure 5) and exhibit the typical bands of a kefiran-based polysaccharide matrix. The broad bands centered at 3292–3303 cm^−1^, corresponding to O–H stretching, are characteristic of the hydroxyl groups of kefiran [15,66], adsorbed water [25], and the phenolic/sugar compounds present in GPE [77]. The shift of these bands toward higher wavenumbers, from 3292 (3K) to 3297, 3299, and 3303 cm^−1^ in 3K-0.5GPE, 3K-1.0GPE, and 3K-1.5GPE, respectively, as the extract concentration increases, indicates a weakening of hydrogen bonds, possibly due to the incorporation of phenolic compounds from the pomace, which induces a reorganization of the hydrophilic interaction network of kefiran. This effect has been documented in corn starch/κ-carrageenan films incorporated with ethanolic grape seed extract [78] and in cellulose/grape seed extract matrices [75].

As shown in Figure 5, in the 3000–2800 cm^−1^ region, the C–H stretching bands of methyl and methylene groups, characteristic of kefiran, are observed [20]. With the highest extract concentration (3K-1.5GPE), the intensity of the bands at 2924 cm^−1^ and 2855 cm^−1^ increased compared to the other films, a behavior related to a higher aliphatic content due to the contribution of lipid components, such as fatty acids from the extract. These results are consistent with the findings of Hasheminya et al. [25], who observed the intensification of the bands at 2927 and 2848 cm^−1^ as the concentration of *Satureja khuzestanica* essential oil, rich in lipid compounds, increased in kefiran/carboxymethylcellulose films. Similarly, in alginate films, the bands at 3009 and 2855 cm^−1^ intensified after the addition of raspberry seed oil [79].

Additionally, the signal at 1745 cm^−1^, attributable to the C=O stretching of carbonyl groups typical of esters and carboxylic acids, intensified with the addition of the extract in the 3K-1.5GPE formulation, which could be related to its lipid content [79], suggesting interactions between these components and the kefiran structure. Likewise, the band at 1645 cm^−1^ may be associated with the H–O–H bending of adsorbed water in the films [80].

On the other hand, according to Hasheminya et al. [25], the bands in the 1135–1070 cm^−1^ range are associated with the ring stretching of carbohydrates and with the C–O–C, C–OH, and C–H groups of polysaccharides. The band at ~1024 cm^−1^ (C–O–C/C–O stretching of ethers and alcohols), common in polysaccharides such as kefiran [20], indicates that the glycosidic structure is maintained in the films despite the incorporation of GPE; its high intensity in all formulations supports that the polymer backbone is not altered. However, a slight shift in the wavenumber is observed, increasing with the addition of GPE from 1024 cm^−1^ (3K) to 1028, 1029, and 1030 cm^−1^ (3K-0.5GPE, 3K-1.0GPE, and 3K-1.5GPE, respectively), suggesting possible interactions between the hydroxyl groups of kefiran and the phenolic compounds. This same behavior has been described in κ-carrageenan matrices, where the addition of pomegranate peel polyphenols increased the wavenumber from 1029 to 1033 cm^−1^ [81].

Finally, the band at 897 cm^−1^, associated with the β-anomeric configuration of glycosidic bonds in polysaccharides such as kefiran [50], did not show significant changes in position or intensity after the incorporation of GPE. This indicates that the primary structure of kefiran remained intact and that interactions with the phenols in the extract did not affect either the glycosidic bonds or the local conformation of the biopolymer. Similar results have been reported in sodium alginate films with grape pomace extract [42], where this band also showed no shifts.

##### Thickness, Tensile Strength, and Elongation

The thickness of the kefiran-based films ranged from 0.052 ± 0.004 mm to 0.133 ± 0.010 mm (Table 4) and increased significantly as the GPE concentration in the films increased (*p* ≤ 0.05). This increase is attributed to the higher solid content provided by GPE, which raises the total volume of the film [82]. Regarding the mechanical properties, the tensile strength values ranged from 3.91 ± 0.69 MPa (3K-1.5GPE) to 9.48 ± 1.61 MPa (3K), while elongation at break ranged from 40.82 ± 8.72% (3K-0.5GPE) to 104.68 ± 14.13% (3K-1.5GPE; Table 4). These results are consistent with those reported by Ghasemlou et al. [22] (5–11 MPa and 40–162%, respectively) and Pop et al. [67] (5.45–8.46 MPa and 65.85–95.89%, respectively), who developed polymeric films based on kefiran.

In general, the addition of GPE caused a decrease (*p* ≤ 0.05) in tensile strength in all treatments and an increase (*p* ≤ 0.05) in elongation at break in the 3K-1.0GPE and 3K-1.5GPE films as the concentration of this component in the formulation increased (Table 4), suggesting a plasticizing effect [78]. This behavior can be explained by the intercalation of phenolic compounds from GPE between kefiran chains, weakening their cohesive interactions and enhancing the mobility of the polymer matrix; this interpretation is consistent with the changes observed in the FT-IR, mainly in the O–H region (Figure 5), indicating modifications in the hydrogen-bonding network of the polysaccharide in the presence of the extract [40,78,83]. The plasticizing effect of the phenolic compounds observed in our study has also been previously reported in starch/κ-carrageenan matrices, where the incorporation of ethanolic grape seed extract reduced tensile strength from 9.07 to 3.50 MPa and increased elongation from 22.37 to 36.87% [78]. Similarly, the addition of pomace anthocyanins to whey protein/κ-carrageenan films slightly decreased tensile strength (from 7.47 to 6.97 MPa) and increased elongation (from 27.74 to 32.36%) [84]. Consistently, Hedayati Rad et al. [30] found that phenolic compounds from rosemary oil improved the flexibility of kefiran/aqueous polyurethane films by reducing molecular interactions within the polymer matrix.

However, the films with the lowest extract concentration (3K-0.5GPE) showed the lowest elongation values, although without a significant difference (*p* > 0.05) compared to the control (3K). This behavior has been previously reported by Ramírez-Tapias et al. [85] in kefiran films plasticized with glycerol, where the lowest concentration of plasticizer (10%) caused a decrease in elongation compared to the control without glycerol, with increases observed from 20% onwards. The authors attributed this initial effect to an anti-plasticization phenomenon, which occurs when low concentrations of plasticizers form relatively strong localized interactions with the polymer (mainly hydrogen bonds), restricting chain mobility and reducing flexibility [86]. Similarly, in films incorporated with GPE, a low concentration (0.5%) could induce an anti-plasticizing effect, whereas increasing the extract content (1.0 and 1.5%) would favor the plasticizing behavior, enhancing matrix mobility and increasing elongation at break [83].

##### Water Solubility

**Figure 6:**
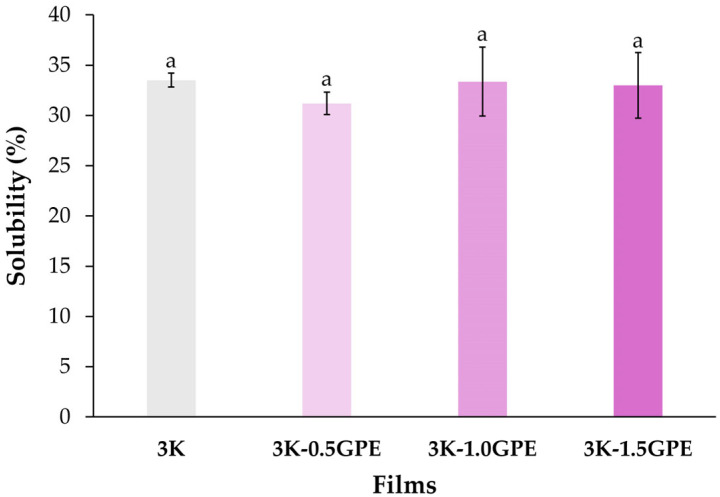
Water solubility (%) of kefiran-based films containing GPE at different concentrations (0, 0.5, 1.0, and 1.5%). Means with the same letters are not significantly different (*p* ≤ 0.05).

Solubility is a critical parameter for materials intended for food packaging applications, as low water solubility prevents dissolution during use [87]. In this study, no significant differences (*p* > 0.05) were observed among the solubility values of the 3K films and those incorporated with GPE (3K-0.5GPE, 3K-1.0GPE, and 3K-1.5GPE), which ranged from 31.18 to 33.50% (Figure 6). These findings are consistent with those reported by Motedayen et al. [26] (28.42%) and Pop et al. [67] (30.32–38.51%) in kefiran-based polymeric films. The results suggest that GPE concentrations (0.5, 1.0, and 1.5%) do not alter the organization of kefiran chains [88]. The phenolic compounds from GPE appear to interact with the polymeric network without increasing the soluble fraction, while maintaining similar solubility in the films (Figure 6); however, such interactions could influence their mechanical properties.

A similar behavior has been described in various polymeric matrices enriched with plant extracts rich in phenolic compounds. Kuan et al. [87] reported solubility values of 75.57–79.74% in alginate films containing mulberry leaf extract (*Morus australis*, 1–4%), without a significant effect from extract addition. Likewise, Goiana et al. [37] observed no changes in the solubility of corn starch films incorporated with GPE (74.1–79.3%) and reported a comparable behavior when acerola extract was added, with statistically similar values (70.5–79.3%). Consistently, Amadeu et al. [51] found no differences in the solubility of pectin films, which were fully soluble in water, after the addition of *Ilex paraguariensis* extract. Similarly, Choi et al. [88] showed that the incorporation of apple peel extract (1, 1.5, and 2%) did not modify the solubility of carboxymethyl cellulose films (47.41%).

##### Water Vapor Permeability (WVP)

**Figure 7:**
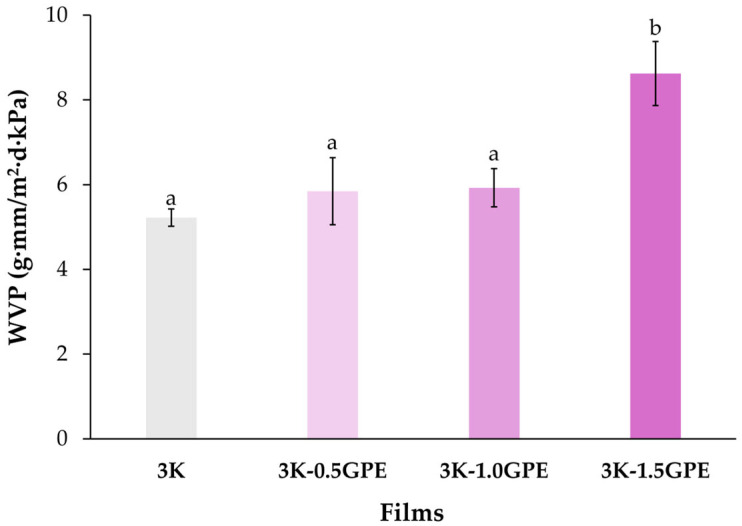
Water vapor permeability of kefiran-based films incorporating GPE at different concentrations (0, 0.5, 1.0, and 1.5%). Means with different letters are significantly different (*p* ≤ 0.05).

WVP is a fundamental property of food packaging, as it indicates its ability to limit moisture transfer from the environment, thereby contributing to food stability and extending shelf life [83]. In this study, the WVP of the films ranged from 5.22 ± 0.20 g·mm/m^2^·d·kPa for the control without extract (3K) to 8.62 ± 0.75 g·mm/m^2^·d·kPa for the film containing 1.5% GPE (3K-1.5GPE; Figure 7), showing an increase as the GPE concentration in the formulation increased. This behavior has also been reported in films based on whey protein, κ-carrageenan, and grape pomace anthocyanins [84]. In general, the increase in the permeability values of the films with higher GPE concentrations is associated with a plasticizing effect of the phenolic compounds present in the extract, which reduces the interactions among kefiran polymer chains, thereby increasing their mobility and facilitating water vapor diffusion through the material [37]. This is consistent with the shifts observed in the O–H region of the FTIR spectra (Figure 5), which suggest a weakening or lower density of hydrogen bonds between polymer chains [78], as discussed in previous sections (Thickness, Tensile Strength, and Elongation and Water Solubility). Furthermore, these changes could reflect a slight reduction in the internal cohesion of the polymer in the presence of higher amounts of GPE, contributing to the observed increase in WVP [89].

According to the literature, similar values have been reported in various polymeric matrices enriched with polyphenolic extracts, such as corn starch/κ-carrageenan with ethanolic extract of grape seed pomace (Wang et al. [78]; 1.08–1.58 g·mm/m^2^·d·kPa) and starch with yerba mate extract (Knapp et al. [53]; 5.95–9.08 g·mm/m^2^·d·kPa), with an increase in WVP observed as the concentration of phenolic extracts in the films increased.

#### 3.5.2. Bioactive Properties of the Kefiran/GPE-Based Films

##### Antioxidant Capacity of the Films

Regarding antioxidant activity, all formulations showed antioxidant properties, with values ranging from 9.87 ± 1.07 to 89.15 ± 2.53 µmol TE/g (ABTS) and 5.83 ± 0.89 to 56.67 ± 1.57 µmol TE/g (DPPH; Table 5). Consistently, the antioxidant activity obtained by DPPH was lower compared to that obtained using ABTS, as the latter method responds to both hydrophilic and lipophilic compounds, whereas DPPH is more selective toward lipophilic or slightly polar molecules [90]. The activity observed in the 3K film suggests that kefiran possesses a slight antioxidant capacity, as previously reported [13]. In contrast, films incorporated with GPE (3K-0.5GPE, 3K-1.0GPE, and 3K-1.5GPE) exhibited significantly higher antioxidant activity (*p* ≤ 0.05) than the control (3K), with antioxidant capacity increasing progressively as the GPE concentration in the formulation increased. This behavior is attributed to the presence of phenolic compounds, such as hydroxycinnamic and hydroxybenzoic acids, flavonoids, stilbenes, and tannins, among others [91]. Moreover, this effect was also evidenced in the total phenolic content of the films (6.33 ± 0.61 to 16.62 ± 1.14 mg GAE/g; Table 5), which increased proportionally with the GPE concentration.

Several studies have documented the antioxidant capacity of polymeric matrices enriched with polyphenols. Gorrasi et al. [41] reported an antioxidant activity of ≈ 35 mg TE/g (ABTS) in films based on alginate/pectin incorporated with Negroamaro grape pomace extract. Marudova et al. [92] found an antioxidant activity of 16.54 µmol TE/g (DPPH) in hydroxypropyl methylcellulose films containing grape seed oil. Martins et al. [93] developed alginate/*Arthrospira* sp. extract films that showed activities of 1537.50 and 190.75 µM TE/mg (ABTS and DPPH, respectively). Similarly, de Moraes et al. [94] reported that the incorporation of papaya peel powder into gelatin films increased antioxidant activity (0.66–1.44 µmol TE/g, DPPH). Likewise, Nguyen [95] observed that chitosan films with gallic acid (0.5 mM) exhibited an increase in antioxidant activity (1.743–2.218 mM TE/g, DPPH), demonstrating that the addition of phenolic compounds enhances this property. Overall, these findings indicate that the incorporation of phenolic extracts into biopolymer matrices is an effective strategy for developing active films with potential applications in food packaging.

##### Antimicrobial Activity of the Films

Regarding antimicrobial activity, the 3K-0.5GPE formulation showed inhibition against *L. monocytogenes*, with a zone of 7.50 ± 0.50 mm, whereas the 3K-1.0GPE and 3K-1.5GPE films did not exhibit activity against this bacterium. Although GPE exhibited activity against *E. coli* (Table 2), the films incorporated with this extract did not show any inhibitory effect against this microorganism (Table 6).

The behavior observed in the antimicrobial activity test is consistent with that reported by Mauro et al. [96] in chitosan-based films incorporated with grape seed oil extract (GSO), where films with the lowest extract dose (chitosan + 0.5 mL GSO) showed higher antimicrobial activity compared to films with the highest dose (chitosan + 1.0 mL GSO) against certain strains of *L. monocytogenes* (DHPS 11B0 and DHPS 13B0) and *E. coli* (ATCC 25922). Similarly, Bruna et al. [44] observed that polylactic acid films incorporated with grape pomace showed lower antimicrobial activity at higher residue concentrations (15%) than at lower concentrations (10%), likely due to slower diffusion of phenolic compounds caused by aggregation of pomace particles within the polymer matrix and the presence of cellulose fibers. This behavior could be attributed to a shielding effect or partial encapsulation phenomenon, which limits the availability of bioactive compounds to diffuse into the medium [96].

In kefiran films incorporated with GPE, the absence of antimicrobial activity could also be related to intermolecular interactions, mainly hydrogen bonds between kefiran and the hydroxyl groups of the phenols, as well as the formation of extract-rich aggregates, which could generate encapsulated regions where the compounds remain trapped and less available to diffuse into the culture medium [97]. These results are consistent with FT-IR analyses (Section Thickness, Tensile Strength, and Elongation), which showed shifts in the bands corresponding to O–H and C–O–C groups, indicative of new intermolecular interactions between the polysaccharide and the phenolic compounds [67,75]. Furthermore, the intensification of these interactions at higher GPE concentrations suggests the formation of a more compact and ordered matrix, restricting the mobility of the phenolic compounds and, therefore, limiting their diffusion into the culture medium and reducing their antimicrobial effectiveness [44,98,99].

Additionally, the lack of activity against *E. coli* could also be due to the higher resistance of this microorganism, attributed to its outer lipopolysaccharide membrane, which acts as a barrier against certain antimicrobial compounds [100]. Overall, these findings suggest that higher concentrations of the active agent do not necessarily translate into greater antimicrobial efficacy, as the polymer matrix may act as a barrier or encapsulating system, limiting the migration and availability of bioactive compounds [43].

## 4. Conclusions

In this study, active films based on the microbial polymer kefiran were developed and enriched with grape pomace extract (GPE) at different concentrations (3K-0.5GPE, 3K-1.0GPE, and 3K-1.5GPE). The incorporation of GPE increased the color parameters (L*, a*, b*, and Δ*E*; 15.11–35.98), thickness (0.052–0.133 mm), and improved UV-Vis light protection as the extract concentration in the formulation increased. FT-IR analysis revealed that the phenolic compounds from GPE established intermolecular interactions with kefiran without altering the glucosidic structure of the polymer. Regarding mechanical properties, a decrease in tensile strength (9.48–3.91 MPa) and an increase in elongation at break (40.82–58.03%) were observed as the GPE concentration increased in the formulations, suggesting a plasticizing effect of the extract. Solubility was not affected by the incorporation of the extract (31.18–33.50%), whereas the water vapor permeability increased with 1.5% GPE incorporation (from 5.22 ± 0.20 to 8.62 ± 0.75 g·mm/m^2^·d·kPa). Moreover, the films exhibited remarkable antioxidant activity in all formulations containing the extract, as well as antimicrobial activity against *L. monocytogenes* in the 3K-0.5GPE formulation. These results suggest that kefiran-based films containing GPE possess properties that make them a potential, sustainable alternative for active food packaging. Their potential ability to preserve the quality characteristics of food products addresses the growing demand for more efficient, safer preservation systems while promoting the use of renewable, biodegradable materials derived from agro-industrial residues.

## Figures and Tables

**Table 1:** Chemical composition of film-forming solutions.

Formulation	Kefiran (%)	Tween 80 (%)	Glycerol (%)	GPE (%)
3K	3.00	0.05	0.75	0.0
3K-0.5GPE	3.00	0.05	0.75	0.5
3K-1.0GPE	3.00	0.05	0.75	1.0
3K-1.5GPE	3.00	0.05	0.75	1.5

K: Kefiran; GPE: Grape pomace extract.

**Table 2:** Antioxidant and antimicrobial activities of the grape pomace extract.

Antioxidant Activity and Total Phenolic Content			Antimicrobial Activity and Total Phenolic Content			
ABTS (µmol TE/g)	DPPH (µmol TE/g)	Total Phenolics (mg GAE/g)	*E. coli*		*L. monocytogenes*	
			MIC (mg/mL)	MBC (mg/mL)	MIC (mg/mL)	MBC (mg/mL)
1081.11 ± 26.73	819.46 ± 10.57	21.65 ± 1.92	50.00	50.00	6.25	25.00

TE: Antioxidant activity expressed as Trolox equivalents; GAE: Total phenolic content expressed as gallic acid equivalents; MIC: Minimum inhibitory concentration; MBC: Minimum bactericidal concentration.

**Table 3:** Color parameters of kefiran-based films containing different concentrations of GPE.

Films	L*	a*	b*	Δ*E*
3K	90.09 ± 0.64 ^a^	−0.66 ± 0.03 ^a^	−4.22 ± 0.21 ^a^	0.00 **
3K-0.5GPE	75.38 ± 0.91 ^b^	1.70 ± 0.09 ^b^	−1.68 ± 0.09 ^b^	15.11 ± 0.92 ^a^
3K-1.0GPE	62.50 ± 0.42 ^c^	4.47 ± 0.08 ^d^	0.22 ± 0.04 ^c^	28.42 ± 0.43 ^b^
3K-1.5GPE	55.07 ± 1.67 ^d^	4.20 ± 0.18 ^c^	2.46 ± 0.15 ^d^	35.98 ± 1.71 ^c^

** Δ*E* values were calculated relative to the extract-free film (3K). Data are expressed as means ± standard deviation. Different superscripts letters within the same column indicate significant differences (*p* ≤ 0.05). K: Kefiran; GPE: Grape pomace extract.

**Table 4:** Physicomechanical properties of kefiran-based films with different concentrations of GPE.

Films	Thickness (mm)	Tensile Strength (MPa)	Elongation at Break (%)
3K	0.052 ± 0.004 ^a^	9.48 ± 1.61 ^a^	58.03 ± 14.91 ^a^
3K-0.5GPE	0.069 ± 0.006 ^b^	5.59 ± 1.14 ^b^	40.82 ± 8.72 ^a^
3K-1.0GPE	0.089 ± 0.007 ^c^	5.29 ± 0.35 ^b^	95.02 ± 16.48 ^b^
3K-1.5GPE	0.113 ± 0.010 ^d^	3.91 ± 0.69 ^c^	104.68 ± 14.13 ^b^

Means with different superscripts in the same column indicate significant differences (*p* ≤ 0.05). K: Kefiran; GPE: Grape pomace extract.

**Table 5:** Antioxidant capacity and total phenolic content of kefiran-based films incorporating GPE at different concentrations.

Films	ABTS (µmol TE/g)	DPPH (µmol TE/g)	Total Phenolics (mg GAE/g)
3K	9.87 ± 1.07 ^a^	5.83 ± 0.89 ^a^	1.00 ± 0.06 ^a^
3K-0.5GPE	51.72 ± 4.48 ^b^	34.56 ± 1.21 ^b^	6.33 ± 0.61 ^b^
3K-1.0GPE	66.11 ± 2.37 ^c^	41.27 ± 1.60 ^c^	12.31 ± 0.82 ^c^
3K-1.5GPE	89.15 ± 2.53 ^d^	56.67 ± 1.57 ^d^	16.62 ± 1.14 ^d^

TE: Trolox equivalents; GAE: Gallic acid equivalents; K: Kefiran; GPE: Grape pomace extract. Means with different superscript letters within a column indicate significant differences (*p* ≤ 0.05).

**Table 6:** Antimicrobial activity of kefiran-based films incorporating GPE at different concentrations.

	Inhibition Zone Diameter (mm)	
Films	*E. coli*	*L. monocytogenes*
3K	n.d.^a^	n.d.^a^
3K-0.5GPE	n.d.^a^	7.50 ± 0.50 ^b^
3K-1.0GPE	n.d.^a^	n.d.^a^
3K-1.5GPE	n.d.^a^	n.d.^a^
Control (−) *	n.d.^a^	n.d.^a^
Control (+) **	17.50 ± 0.71 ^b^	33.00 ± 2.82 ^c^

K: Kefiran; GPE: Grape pomace extract. n.d.: Not detected. * Isotonic saline solution. ** Amoxicillin–clavulanate. Means with different superscript letters indicate significant differences (*p* ≤ 0.05).

## Data Availability

The original contributions presented in this study are included in the article. Further inquiries can be directed at the corresponding author.

## References

[1] Hernández-García E., Vargas M., González-Martínez C., Chiralt A. (2021). Biodegradable Antimicrobial Films for Food Packaging: Effect of Antimicrobials on Degradation. Foods.

[2] Kumar N., Pratibha, Prasad J., Yadav A., Upadhyay A., Neeraj, Shukla S., Petkoska A.T., Heena, Suri S. (2023). Recent Trends in Edible Packaging for Food Applications—Perspective for the Future. Food Eng. Rev..

[3] Dirpan A., Ainani A.F., Djalal M. (2023). A Review on Biopolymer-Based Biodegradable Film for Food Packaging: Trends over the Last Decade and Future Research. Polymers.

[4] Khan N., Sudhakar K., Mamat R. (2025). Biodegradable Plastics from Marine Biomass: A Solution to Marine Plastic Pollution. J. Hazard. Mater. Adv..

[5] Cazón P., Velazquez G., Ramírez J.A., Vázquez M. (2017). Polysaccharide-Based Films and Coatings for Food Packaging: A Review. Food Hydrocoll..

[6] Khodaei D., Álvarez C., Mullen A.M. (2021). Biodegradable Packaging Materials from Animal Processing Co-Products and Wastes: An Overview. Polymers.

[7] Prakoso F.A.H., Indiarto R., Utama G.L. (2023). Edible Film Casting Techniques and Materials and Their Utilization for Meat-Based Product Packaging. Polymers.

[8] Shahidi F., Hossain A. (2022). Preservation of Aquatic Food Using Edible Films and Coatings Containing Essential Oils: A Review. Crit. Rev. Food Sci. Nutr..

[9] Matloob A., Ayub H., Mohsin M., Ambreen S., Khan F.A., Oranab S., Rahim M.A., Khalid W., Nayik G.A., Ramniwas S. (2023). A Review on Edible Coatings and Films: Advances, Composition, Production Methods, and Safety Concerns. ACS Omega.

[10] Ribeiro I.S., Maciel G.M., Bortolini D.G., Fernandes I.A.A., Maroldi W.V., Pedro A.C., Rubio F.T.V., Haminiuk C.W.I. (2024). Sustainable Innovations in Edible Films and Coatings: An Overview. Trends Food Sci. Technol..

[11] Díaz-Montes E., Castro-Muñoz R. (2021). Edible Films and Coatings as Food-Quality Preservers: An Overview. Foods.

[12] Antonino C., Difonzo G., Faccia M., Caponio F. (2024). Effect of Edible Coatings and Films Enriched with Plant Extracts and Essential Oils on the Preservation of Animal-Derived Foods. J. Food Sci..

[13] de Carvalho A.P.A., Conte-Junior C.A. (2021). Food-Derived Biopolymer Kefiran Composites, Nanocomposites and Nanofibers: Emerging Alternatives to Food Packaging and Potentials in Nanomedicine. Trends Food Sci. Technol..

[14] Marangoni Júnior L., Vieira R.P., Rodrigues Anjos C.A. (2020). Kefiran-Based Films: Fundamental Concepts, Formulation Strategies and Properties. Carbohydr. Polym..

[15] Radhouani H., Gonçalves C., Maia F.R., Oliveira J.M., Reis R.L. (2018). Kefiran Biopolymer: Evaluation of Its Physicochemical and Biological Properties. J. Bioact. Compat. Polym..

[16] Tan K.X., Chamundeswari V.N., Loo S.C.J. (2020). Prospects of Kefiran as a Food-Derived Biopolymer for Agri-Food and Biomedical Applications. RSC Adv..

[17] Van Wyk J., Grumezescu A.M., Holban A.M. (2019). Kefir: The Champagne of Fermented Beverages. Fermented Beverages.

[18] Moradi Z., Kalanpour N. (2019). Kefiran, a Branched Polysaccharide: Preparation, Properties and Applications: A Review. Carbohydr. Polym..

[19] Piermaria J.A., Pinotti A., Garcia M.A., Abraham A.G. (2009). Films Based on Kefiran, an Exopolysaccharide Obtained from Kefir Grain: Development and Characterization. Food Hydrocoll..

[20] Piermaria J., Bosch A., Pinotti A., Yantorno O., Garcia M.A., Abraham A.G. (2011). Kefiran Films Plasticized with Sugars and Polyols: Water Vapor Barrier and Mechanical Properties in Relation to Their Microstructure Analyzed by ATR/FT-IR Spectroscopy. Food Hydrocoll..

[21] Ghasemlou M., Khodaiyan F., Oromiehie A. (2011). Physical, Mechanical, Barrier, and Thermal Properties of Polyol-Plasticized Biodegradable Edible Film Made from Kefiran. Carbohydr. Polym..

[22] Ghasemlou M., Khodaiyan F., Oromiehie A., Yarmand M.S. (2011). Development and Characterisation of a New Biodegradable Edible Film Made from Kefiran, an Exopolysaccharide Obtained from Kefir Grains. Food Chem..

[23] Motedayen A.A., Khodaiyan F., Salehi E.A. (2013). Development and Characterisation of Composite Films Made of Kefiran and Starch. Food Chem..

[24] Sabaghi M., Maghsoudlou Y., Habibi P. (2015). Enhancing Structural Properties and Antioxidant Activity of Kefiran Films by Chitosan Addition. Food Struct..

[25] Hasheminya S.M., Mokarram R.R., Ghanbarzadeh B., Hamishekar H., Kafil H.S., Dehghannya J. (2019). Development and Characterization of Biocomposite Films Made from Kefiran, Carboxymethyl Cellulose and *Satureja khuzestanica* Essential Oil. Food Chem..

[26] Motedayen A.A., Khodaiyan F., Salehi E.A., Hosseini S. (2019). Characterisation of Biocomposite Film Made of Kefiran and Carboxymethyl Cellulose (CMC). J. Food Bioprocess Eng..

[27] Salmanian H., Khodaiyan F., Hosseini S.S. (2019). Biodegradable Kefiran-Chitosan-Nanocellulose Blend Film: Production and Physical, Barrier, Mechanical, Thermal, and Structural Properties. J. Food Bioprocess Eng..

[28] Zolfi M., Khodaiyan F., Mousavi M., Hashemi M. (2014). The Improvement of Characteristics of Biodegradable Films Made from Kefiran-Whey Protein by Nanoparticle Incorporation. Carbohydr. Polym..

[29] Zolfi M., Khodaiyan F., Mousavi M., Hashemi M. (2014). Development and Characterization of the Kefiran-Whey Protein Isolate-TiO_2_ Nanocomposite Films. Int. J. Biol. Macromol..

[30] Hedayati Rad F., Sharifan A., Asadi G. (2018). Physicochemical and Antimicrobial Properties of Kefiran/Waterborne Polyurethane Film Incorporated with Essential Oils on Refrigerated Ostrich Meat. LWT.

[31] Jourabian F., Nouri M. (2023). Optimization of Formulated Kefiran/*Malva Neglecta* Film with Rice Bran Oil to Maintain Cauliflower Quality in Storage. Proc. Natl. Acad. Sci. India Sect. B Biol. Sci..

[32] Pinto T., Pinto A., Vilela A. (2023). Edible Coatings and Films for Preparation of Grapevine By-Product Infusions and in Freshly Processed Products. Coatings.

[33] Benković M., Cigić F., Valinger D., Sokač Cvetnić T., Jurinjak Tušek A., Jurina T., Gajdoš Kljusurić J., Radojčić Redovniković I. (2024). Towards Wine Waste Reduction: Up-Cycling Wine Pomace into Functional Fruit Bars. Processes.

[34] Wang C., You Y., Huang W., Zhan J. (2024). The High-Value and Sustainable Utilization of Grape Pomace: A Review. Food Chem. X.

[35] Peixoto C.M., Dias M.I., Alves M.J., Calhelha R.C., Barros L., Pinho S.P., Ferreira I.C.F.R. (2018). Grape Pomace as a Source of Phenolic Compounds and Diverse Bioactive Properties. Food Chem..

[36] Singh A.K., Kim J.Y., Lee Y.S. (2022). Phenolic Compounds in Active Packaging and Edible Films/Coatings: Natural Bioactive Molecules and Novel Packaging Ingredients. Molecules.

[37] Goiana M.L., de Freitas Rosa M., Mattos A.L.A., Fernandes F.A.N. (2025). Development of Plasma-Treated Corn-Starch-Based Film Incorporated with Acerola and Grape Pomace Extract Possessing pH-Sensing Capability. Polymers.

[38] Xu Y., Scales A., Jordan K., Kim C., Sismour E. (2017). Starch Nanocomposite Films Incorporating Grape Pomace Extract and Cellulose Nanocrystal. J. Appl. Polym. Sci..

[39] Ferreira A.S., Nunes C., Castro A., Ferreira P., Coimbra M.A. (2014). Influence of Grape Pomace Extract Incorporation on Chitosan Films Properties. Carbohydr. Polym..

[40] Xu Y., Willis S., Jordan K., Sismour E. (2018). Chitosan Nanocomposite Films Incorporating Cellulose Nanocrystals and Grape Pomace Extracts. Packag. Technol. Sci..

[41] Gorrasi G., Viscusi G., Gerardi C., Lamberti E., Giovinazzo G. (2022). Physicochemical and Antioxidant Properties of White (*Fiano cv*) and Red (*Negroamaro cv*) Grape Pomace Skin Based Films. J. Polym. Environ..

[42] Gubitosa J., Rizzi V., Marasciulo C., Maggi F., Caprioli G., Mustafa A.M., Fini P., De Vietro N., Aresta A.M., Cosma P. (2023). Realizing Eco-Friendly Water-Resistant Sodium-Alginate-Based Films Blended with a Polyphenolic Aqueous Extract from Grape Pomace Waste for Potential Food Packaging Applications. Int. J. Mol. Sci..

[43] Deng Q., Zhao Y. (2011). Physicochemical, Nutritional, and Antimicrobial Properties of Wine Grape (cv. Merlot) Pomace Extract-Based Films. J. Food Sci..

[44] Bruna J.E., Castillo M., López de Dicastillo C., Muñoz-Shugulí C., Lira M., Guarda A., Rodríguez-Mercado F.J., Galotto M.J. (2023). Development of Active Biocomposite Films Based on Poly (lactic acid) and Wine by-Product: Effect of Grape Pomace Content and Extrusion Temperature. J. Appl. Polym. Sci..

[45] da Silva D.J., de Oliveira M.M., Wang S.H., Carastan D.J., Rosa D.S. (2022). Designing Antimicrobial Polypropylene Films with Grape Pomace Extract for Food Packaging. Food Packag. Shelf Life.

[46] Gaber Ahmed G.H., Fernández-González A., Díaz García M.E. (2020). Nano-Encapsulation of Grape and Apple Pomace Phenolic Extract in Chitosan and Soy Protein via Nanoemulsification. Food Hydrocoll..

[47] Guzmán-Díaz D.A., Treviño-Garza M.Z., Rodríguez-Romero B.A., Gallardo-Rivera C.T., Amaya-Guerra C.A., Báez-González J.G. (2019). Development and Characterization of Gelled Double Emulsions Based on Chia (*Salvia hispanica* L.) Mucilage Mixed with Different Biopolymers and Loaded with Green Tea Extract (*Camellia sinensis*). Foods.

[48] Treviño-Garza M.Z., Yañez-Echeverría S.A., García S., Mora-Zúñiga A.E., Arévalo-Niño K. (2020). Physico-Mechanical, Barrier and Antimicrobial Properties of Linseed Mucilage Films Incorporated with *H. Virginiana* Extract. Rev. Mex. Ing. Quím..

[49] Drosou C., Kyriakopoulou K., Bimpilas A., Tsimogiannis D., Krokida M. (2015). A Comparative Study on Different Extraction Techniques to Recover Red Grape Pomace Polyphenols from Vinification Byproducts. Ind. Crops Prod..

[50] Montoille L., Morales Vicencio C., Fontalba D., Ortiz J.A., Moreno-Serna V., Peponi L., Matiacevich S., Zapata P.A. (2021). Study of the Effect of the Addition of Plasticizers on the Physical Properties of Biodegradable Films Based on Kefiran for Potential Application as Food Packaging. Food Chem..

[51] Amadeu C.A.A., Silva F.B., Souza C.J.F., Koschevic M.T., Schoeninger V., Falcão E.A., Garcia V.A.D.S., Cardoso C.A.L., Martelli S.M. (2024). Pectin Edible Films Filled with *Ilex Paraguariensis* Concentrate Extract and Its Characterization. Polymers.

[52] Moges T.G., Kassa H.S., Woldemariam H.W. (2024). Optimization and Characterization of Active Bio-Plastic Film from Tamarind (*Tamarindus indica* L.) Seed Starch Enriched with Red Grape Pomace Extract. Biomass Convers. Biorefin..

[53] Knapp M.A., dos Santos D.F., Pilatti-Riccio D., Deon V.G., dos Santos G.H.F., Pinto V.Z. (2019). Yerba Mate Extract in Active Starch Films: Mechanical and Antioxidant Properties. J. Food Process. Preserv..

[54] Xu Y., Burton S., Kim C., Sismour E. (2016). Phenolic Compounds, Antioxidant, and Antibacterial Properties of Pomace Extracts from Four Virginia-Grown Grape Varieties. Food Sci. Nutr..

[55] Ky I., Lorrain B., Kolbas N., Crozier A., Teissedre P.L. (2014). Wine By-Products: Phenolic Characterization and Antioxidant Activity Evaluation of Grapes and Grape Pomaces from Six Different French Grape Varieties. Molecules.

[56] Ky I., Teissedre P.L. (2015). Characterisation of Mediterranean Grape Pomace Seed and Skin Extracts: Polyphenolic Content and Antioxidant Activity. Molecules.

[57] Moutinho J., Gouvinhas I., Domínguez-Perles R., Barros A. (2023). Optimization of the Extraction Methodology of Grape Pomace Polyphenols for Food Applications. Molecules.

[58] Batista D., de Matuoka e Chiocchetti G., Macedo J.A. (2023). Effect of Enzymatic Biotransformation on the Hypotensive Potential of Red Grape Pomace Extract. Foods.

[59] Chengolova Z., Ivanov Y., Godjevargova T. (2023). Comparison of Identification and Quantification of Polyphenolic Compounds in Skins and Seeds of Four Grape Varieties. Molecules.

[60] Rasines-Perea Z., Ky I., Cros G., Crozier A., Teissedre P.L. (2018). Grape Pomace: Antioxidant Activity, Potential Effect Against Hypertension and Metabolites Characterization after Intake. Diseases.

[61] Cheng V.J., Bekhit A.E.-D.A., McConnell M., Mros S., Zhao J. (2012). Effect of Extraction Solvent, Waste Fraction and Grape Variety on the Antimicrobial and Antioxidant Activities of Extracts from Wine Residue from Cool Climate. Food Chem..

[62] Silva V., Igrejas G., Falco V., Santos T.P., Torres C., Oliveira A.M.P., Pereira J.E., Amaral J.S., Poeta P. (2018). Chemical Composition, Antioxidant and Antimicrobial Activity of Phenolic Compounds Extracted from Wine Industry by-Products. Food Control.

[63] Grosu A.C., Diguță F.C., Pristavu M.-C., Popa A., Badea F., Dragoi Cudalbeanu M., Orțan A., Dopcea I., Băbeanu N. (2024). Exploring the Phytochemical Profiles, and Antioxidant and Antimicrobial Activities of the Hydroethanolic Grape Pomace Extracts from Two Romanian Indigenous Varieties. Fermentation.

[64] Hassan Y.I., Kosir V., Yin X., Ross K., Diarra M.S. (2019). Grape Pomace as a Promising Antimicrobial Alternative in Feed: A Critical Review. J. Agric. Food Chem..

[65] Martins E.F., de Moura N.K., de Moura T.K., de Araújo T.V., Machado J.P.B., Passador F.R., Esposito E. (2022). Determination and Standardization of the Kefiran Extraction Protocol for Possible Pharmacological Applications. Carbohydr. Polym. Technol. Appl..

[66] Pop C.R., Salanță L.C., Rotar A.M., Semeniuc C.A. (2016). Influence of Extraction Conditions on Characteristics of Microbial Polysaccharide Kefiran Isolated from Kefir Grains Biomass. J. Food Nutr. Res..

[67] Pop C.R., Coldea T.E., Salanţă L.C., Nistor A.L., Borşa A., Fărcaș A.C., Florian V.C., Rotar A.M. (2021). The Effect of Extraction Conditions on the Barrier and Mechanical Properties of Kefiran Films. Coatings.

[68] Nogueira G.F., Soares I.H.B.T., Soares C.T., Fakhouri F.M., de Oliveira R.A. (2022). Development and Characterization of Arrowroot Starch Films Incorporated with Grape Pomace Extract. Polysaccharides.

[69] Etxabide A., Yang Y., Maté J.I., de la Caba K., Kilmartin P.A. (2022). Developing Active and Intelligent Films through the Incorporation of Grape Skin and Seed Tannin Extracts into Gelatin. Food Packag. Shelf Life.

[70] Mileti O., Baldino N., Filice F., Lupi F.R., Sinicropi M.S., Gabriele D. (2023). Formulation Study on Edible Film from Waste Grape and Red Cabbage. Foods.

[71] Pan J., Li C., Liu J., Jiao Z., Zhang Q., Lv Z., Yang W., Chen D., Liu H. (2024). Polysaccharide-Based Packaging Coatings and Films with Phenolic Compounds in Preservation of Fruits and Vegetables—A Review. Foods.

[72] Schmitz L., Moura S. (2025). Grape By-Products for Smart Packaging Films: Antioxidant, pH-Sensitive, UV-Blocking and Antimicrobial Properties for Food Preservation. Packag. Technol. Sci..

[73] Qiu Z., Niu W., Wang S., Yu F., Yu Y., Fan J., Zheng L., Wang Y., Xiao Z., Xie Y. (2021). Multifunctional Composite Film Based on Biodegradable Grape Skin and Polyvinyl Alcohol. Cellulose.

[74] Priyadarshi R., Kim S.M., Rhim J.W. (2021). Carboxymethyl Cellulose-Based Multifunctional Film Combined with Zinc Oxide Nanoparticles and Grape Seed Extract for the Preservation of High-Fat Meat Products. Sustain. Mater. Technol..

[75] Ji X., Xu Z., Xia X., Wei Z., Zhang J., Xia G., Ji X. (2023). Cellulose/Grape-Seed-Extract Composite Films with High Transparency and Ultraviolet Shielding Performance Fabricated from Old Cotton Textiles. Polymers.

[76] Botalo A., Inprasit T., Ummartyotin S., Chainok K., Vatthanakul S., Pisitsak P. (2024). Smart and UV-Resistant Edible Coating and Films Based on Alginate, Whey Protein, and Curcumin. Polymers.

[77] Silva J.T.d.P., Borges M.H., de Souza C.A.C., Fávaro-Trindade C.S., Sobral P.J.d.A., de Oliveira A.L., Martelli-Tosi M. (2024). Grape Pomace Rich-Phenolics and Anthocyanins Extract: Production by Pressurized Liquid Extraction in Intermittent Process and Encapsulation by Spray-Drying. Foods.

[78] Wang C., An X., Lu Y., Li Z., Gao Z., Tian S. (2022). Biodegradable Active Packaging Material Containing Grape Seed Ethanol Extract and Corn Starch/κ-Carrageenan Composite Film. Polymers.

[79] Kowalonek J., Łukomska B., Łukomska O., Stachowiak-Trojanowska N. (2024). Alginate Films Enriched in Raspberry and/or Black Currant Seed Oils as Active Food Packaging. Molecules.

[80] Shah Y.A., Bhatia S., Chinnam S., Al-Harrasi A., Tarahi M., Khan T.S., Alam T., Koca E., Aydemir L.Y., Philip A.K. (2024). Myrrh Oleo-Gum Resin as a Functional Additive in Pectin and κ-Carrageenan Composite Films for Food Packaging. Food Sci. Nutr..

[81] Liu Y., Zhang X., Li C., Qin Y., Xiao L., Liu J. (2020). Comparison of the Structural, Physical and Functional Properties of κ-Carrageenan Films Incorporated with Pomegranate Flesh and Peel Extracts. Int. J. Biol. Macromol..

[82] Wang N., Wei J., Wang C., Ren J. (2025). Preparation, Physicochemical Properties, Biological Activity of a Multifunctional Composite Film Based on Zein/Citric Acid Loaded with Grape Seed Extract and Its Application in Solid Lipid Packaging. Foods.

[83] Coma M.E., Peltzer M.A., Delgado J.F., Salvay A.G. (2019). Water Kefir Grains as an Innovative Source of Materials: Study of Plasticiser Content on Film Properties. Eur. Polym. J..

[84] Yekta R., Dabbagh Moghaddam A., Hosseini H., Sharifan A., Hadi S., Hosseini-Shokouh S.J. (2024). Effect of Using Biodegradable Film Constituting Red Grape Anthocyanins as a Novel Packaging on the Qualitative Attributes of Emergency Food Bars during Storage. Food Sci. Nutr..

[85] Ramírez-Tapias Y.A., Rezzani G.D., Delgado J.F., Peltzer M.A., Salvay A.G. (2024). New Materials from the Integral Milk Kefir Grain Biomass and the Purified Kefiran: The Role of Glycerol Content on the Film’s Properties. Polymers.

[86] Mali S., Grossmann M.V.E., Garcia M.A., Martino M.N., Zaritzky N.E. (2008). Antiplasticizing effect of glycerol and sorbitol on the properties of cassava starch films. Braz. J. Food Technol..

[87] Kuan Y.L., Sivanasvaran S.N., Pui L.P., Yusof Y.A., Senphan T. (2020). Physicochemical Properties of Sodium Alginate Edible Film Incorporated with Mulberry (*Morus australis*) Leaf Extract. Pertanika J. Trop. Agric. Sci..

[88] Choi I., Chang Y., Shin S.H., Joo E., Song H.J., Eom H., Han J. (2017). Development of Biopolymer Composite Films Using a Microfluidization Technique for Carboxymethylcellulose and Apple Skin Particles. Int. J. Mol. Sci..

[89] Fitriani F., Aprilia S., Bilad M.R., Arahman N., Usman A., Huda N., Kobun R. (2022). Optimization of Biocomposite Film Based on Whey Protein Isolate and Nanocrystalline Cellulose from Pineapple Crown Leaf Using Response Surface Methodology. Polymers.

[90] Granados-Guzmán G., Salazar-Aranda R., Garza-Tapia M., Castro-Ríos R., Waksman De Torres N. (2017). Optimization and Validation of Two High-Throughput Methods Indicating Antiradical Activity. Curr. Anal. Chem..

[91] Beres C., Costa G.N.S., Cabezudo I., da Silva-James N.K., Teles A.S.C., Cruz A.P.G., Mellinger-Silva C., Tonon R.V., Cabral L.M.C., Freitas S.P. (2017). Towards Integral Utilization of Grape Pomace from Winemaking Process: A Review. Waste Manag..

[92] Marudova M., Sotirov S., Zhelyazkov S., Zsivanovits G. (2021). Formulation and Characterization of Hydroxypropyl Methylcellulose Edible Films Containing Grape Seed Oil. Macromol. Symp..

[93] Martins V.F.R., Poças F., Pintado M., Morais R.M.S.C., Morais A.M.M.B. (2024). Edible Films with Protein and Bioactive Compounds from *Arthrospira* sp. Biol. Life Sci. Forum.

[94] de Moraes Crizel T., de Oliveira Rios A., Alves V.D., Bandarra N., Moldão-Martins M., Hickmann Flôres S. (2018). Biodegradable Films Based on Gelatin and Papaya Peel Microparticles with Antioxidant Properties. Food Bioprocess Technol..

[95] Nguyen T.A. (2023). Characterization of Chitosan-Based Active Film Incorporating with Gallic Acid. Int. J. Multidiscip. Res. Anal..

[96] Mauro M., Pinto P., Settanni L., Puccio V., Vazzana M., Hornsby B.L., Fabbrizio A., Di Stefano V., Barone G., Arizza V. (2022). Chitosan Film Functionalized with Grape Seed Oil—Preliminary Evaluation of Antimicrobial Activity. Sustainability.

[97] Mugnaini G., Bonini M., Gentile L., Panza O., Del Nobile M.A., Conte A., Esposito R., D’Errico G., Moccia F., Panzella L. (2024). Effect of design and molecular interactions on the food-preserving properties of alginate/pullulan edible films loaded with grape pomace extract. J. Food Eng..

[98] Xue F., Zhao M., Liu X., Chu R., Qiao Z., Li C., Adhikari B. (2021). Physicochemical properties of chitosan/zein/essential oil emulsion-based active films functionalized by polyphenols. Future Foods.

[99] Talón E., Trifkovic K.T., Vargas M., Chiralt A., González-Martínez C. (2017). Release of polyphenols from starch–chitosan based films containing thyme extract. Carbohydr. Polym..

[100] Zhou G., Wang Q., Wang Y., Wen X., Peng H., Peng R., Shi Q., Xie X., Li L. (2023). Outer Membrane Porins Contribute to Antimicrobial Resistance in Gram-Negative Bacteria. Microorganisms.

